# Epidemiology of Hypothyroidism, Hyperthyroidism and Positive Thyroid Antibodies in the Croatian Population

**DOI:** 10.3390/biology11030394

**Published:** 2022-03-02

**Authors:** Ivana Strikić Đula, Nikolina Pleić, Mirjana Babić Leko, Ivana Gunjača, Vesela Torlak, Dubravka Brdar, Ante Punda, Ozren Polašek, Caroline Hayward, Tatijana Zemunik

**Affiliations:** 1 Health Center Sibenik, 22 000 Sibenik, Croatia; ivanastrikic.is@gmail.com; 2 Department of Medical Biology, School of Medicine, University of Split, 21 000 Split, Croatia; npleic@mefst.hr (N.P.); mbabic@mefst.hr (M.B.L.); igunjaca@mefst.hr (I.G.); 3 Department of Nuclear Medicine, University Hospital of Split, 21 000 Split, Croatia; veselakbsplit@yahoo.com (V.T.); d.brdar.dr@gmail.com (D.B.); ante.punda@mefst.hr (A.P.); 4 Department of Public Health, School of Medicine, University of Split, 21 000 Split, Croatia; opolasek@gmail.com; 5 MRC Human Genetics Unit, Institute of Genetics and Cancer, University of Edinburgh, Edinburgh EH4 2XU, UK; caroline.hayward@ed.ac.uk

**Keywords:** hypothyroidism, hyperthyroidism, epidemiology, undiagnosed cases, prevalence

## Abstract

**Simple Summary:**

The aim of this paper was to analyse the prevalence of hypothyroidism and hyperthyroidism in the Croatian population. This is the first epidemiological study of its kind conducted in our population. It is important to correctly diagnose thyroid dysfunction due to the detrimental effects of thyroid dysfunction on human health (especially in pregnant women and patients with cardiovascular diseases). The results of our study conducted on 4402 individuals, showed a higher prevalence of hypothyroidism in our country (10.5%) compared to other countries, while the prevalence of hyperthyroidism was quite similar (1.3%). We also observed that a high proportion of thyroid disorders remained undiagnosed (as many as 92.6% subclinical hypothyroid, 93.9% clinical hypothyroid, 83% subclinical hyperthyroid and 71.4% clinical hyperthyroid participants). Therefore, our study indicates that investing in prevention programs is crucial.

**Abstract:**

Thyroid dysfunction appears to be the leading endocrine disorder. We conducted a cross-sectional study on 4402 individuals from three Croatian cohorts. The aim of this study was to analyse the prevalence of diagnosed and undiagnosed hypothyroidism, hyperthyroidism (subclinical and clinical) and positive thyroid antibodies in the Croatian population. The results of the study indicated that 17.6% of participants were euthyroid with positive antibodies. The prevalence of clinical and subclinical hypothyroidism was 3% and 7.4%, respectively, while the prevalence of clinical and subclinical hyperthyroidism was 0.2% and 1.1%, respectively. Among them, 92.6% subclinical hypothyroid, 93.9% clinical hypothyroid, 83% subclinical hyperthyroid and 71.4% clinical hyperthyroid participants were undiagnosed. Finally, the prevalence of undiagnosed subclinical and clinical hypothyroidism in our population was 6.9% and 2.8%, respectively, while the prevalence of undiagnosed subclinical and clinical hyperthyroidism was 0.9% and 0.1%, respectively. Women showed a higher prevalence of thyroid disorders; 1.57 times higher odds of euthyroidism with positive antibodies, 2.1 times higher odds of subclinical hyperthyroidism, 2.37 times higher odds of clinical hypothyroidism and 1.58 times higher odds of subclinical hypothyroidism than men. These results indicate an extremely high proportion of undiagnosed cases, and therefore require investments in a prevention programme.

## 1. Introduction

Thyroid dysfunction affects a significant portion of the population and appears to be the leading endocrine disorder [1]. Until the implementation of the iodine fortification program in Europe, goitre was a significant health problem. Increased iodine intake appears to cause an increase in the prevalence of hypothyroidism [2]. The diagnosis of thyroid dysfunction is mainly based on biochemical parameters, with thyroid-stimulating hormone (TSH) levels as the most sensitive indicator of thyroid status. Accordingly, subclinical hypothyroidism is defined as TSH above and free thyroxine (fT4) within the reference range, while clinical hypothyroidism is defined as TSH above and fT4 under the reference range. Likewise, the inverse hormone pattern defines subclinical hyperthyroidism (TSH under and fT4 within the reference range) and clinical hyperthyroidism (TSH under and fT4 above the reference range) [3].

The American Thyroid Association reported that 20 million Americans have some form of thyroid disease and that more than 12% of the US population will develop a thyroid condition during their lifetime [4]. The National Health and Nutrition Examination Survey (NHANES III) selected a reference US population of 13,344 participants without recognized thyroid disease by determining serum concentrations of TSH, T4, thyroglobulin antibodies (TgAb), and thyroid peroxidase antibodies (TPOAb) [5]. They found that hypothyroidism was present in 4.6% of the population (with a higher presence of subclinical than clinical hypothyroidism), while hyperthyroidism was present in 1.3% of the population (with the almost equal presence of subclinical and clinical hyperthyroidism) [5]. The first meta-analysis of the epidemiology of thyroid dysfunction in European populations was conducted in 2014. The authors stated that the prevalence of undiagnosed thyroid dysfunction was analysed in 7 studies, with an average score of 6.71%, which is 4.94% and 1.72% for undiagnosed hypothyroidism and hyperthyroidism, respectively. The prevalence of both previously diagnosed and undiagnosed thyroid dysfunction was assessed in 9 studies, with an average score of 3.82%, which is 3.05% and 0.75% for hypothyroidism and hyperthyroidism, respectively [6]. Another meta-analysis performed in 2019, which included 20 studies, showed a 4.7% prevalence of undiagnosed total (subclinical plus clinical) hypothyroidism in Europe, with a 0.65% prevalence of undiagnosed clinical hypothyroidism and a 4.11% prevalence of undiagnosed subclinical hypothyroidism [7].

In other populations, there are fewer studies on the prevalence of hypothyroidism and hyperthyroidism, especially in undiagnosed cases. These studies are often limited by the selection of participants. For example, data on the prevalence of diagnosed and undiagnosed cases of thyroid dysfunction among the elderly population in Australia show almost half the number compared to the European population (3.6%) [8]. More than a billion people live in iodine-deficient areas, with the most vulnerable areas being Southeast Asia, South America and Central Africa [3]. Several studies from Brazil have shown different prevalence of clinical and subclinical hypothyroidism, suggesting the existence of environmental and especially ethnic differences because this county has a high ethnic admixture. Sufficient iodine has caused an increased prevalence of hypothyroidism in China in the last decade [9]. Moreover, another Chinese study observed an almost linear association between higher urinary iodine concentrations and an increased risk of Hashimoto’s thyroiditis [10]. The lack of population-based studies of thyroid dysfunction in Africa is the cause of the lack of data on the prevalence in these populations [3].

Several factors such as age, gender, ethnicity and geographical location may affect the prevalence of thyroid dysfunction. Geographical location appears to be associated with dietary iodine intake [11]. The prevalence of hypothyroidism is usually higher in women than in men, among individuals ≥65 years of age and in Eastern and Southern Europe than in Northern and Western Europe [7].

The purpose of this study was to analyse the prevalence of hypothyroidism, hyperthyroidism (subclinical and clinical) and positive thyroid antibodies in the Croatian population and to determine the prevalence of undiagnosed cases. In addition, we analysed the main characteristics of each group.

## 2. Materials and Methods

### 2.1. Study Population

This cross-sectional study was performed on samples from three Croatian populations: the mainland city of Split and the islands of Vis and Korčula. The samples were obtained from the large-scale “10,001 Dalmatians” biobank project [12]. The participants were adult volunteers aged 18–98 years from the general population. Plasma samples were collected and stored at a temperature of −80 ℃. The samples were used to measure thyroid hormone and antibody levels. After excluding participants who had missing data for at least one hormone, the final sample comprised 4402 eligible participants. Written informed consent was obtained from all participants, and the study protocol was approved by the Ethical board of the University of Split, School of Medicine (No: 2181-198-03-04-14-0031 and 2181-198-03-04-19-0022).

### 2.2. Biochemical Measurements

Circulating thyroid hormone and antibody levels in the plasma of participants were determined by immunoassay methods with the Liaison XL Biomedica Chemiluminescence Analyzer, using in vitro assays for quantitation of thyroglobulin (Tg) (REF 311861), TSH (REF 311211), free triiodothyronine (fT3) (REF 311531), fT4 (REF 311611), TgAb (REF 311711) and TPOAb (REF 311701). Study population reference ranges were: Tg 0.2–50 ng/mL, TSH 0.3–3.6 mIU/L, fT3 3.39–6.47 pmol/L, fT4 10.29–21.88 pmol/L, TgAb 5–100 IU/mL and TPOAb levels 1–16 IU/mL. Biochemical measurements were performed in the Biochemistry Laboratory in the Department of Nuclear Medicine at the University Hospital, Split.

### 2.3. Definitions

Euthyroidism was defined as TSH, fT3 and fT4 within the reference ranges and with the absence of positive antibodies. Participants with all three hormones within the reference ranges but with a presence of positive antibodies (positive TPOAb and/or TgAb) were defined as euthyroid with positive antibodies. Subclinical hypothyroidism was defined as TSH > 3.6 mlU/L and fT3 and fT4 within the reference ranges, while clinical hypothyroidism was defined as TSH > 3.6 mlU/L, fT3 ≤ 6.47 pmol/L and fT4 < 10.29 pmol/L. Subclinical hyperthyroidism was defined as TSH < 0.3 mlU/L and fT3 and fT4 within the reference ranges, while clinical hyperthyroidism was defined as TSH < 0.3 mlU/L, fT3 ≥ 3.39 and fT4 > 21.88 pmol/L.

### 2.4. Statistical Analysis

Continuous variables are expressed as means with standard deviations or as medians with lower and upper quartiles, and categorical variables as frequencies (percentages). The χ^2^ test or the Fisher’s exact test for dichotomous variables were used to assess if there were significant differences between the groups. For continuous variables, ANOVA or Kruskal–Wallis tests were used to assess if there were significant differences between the groups. Univariate logistic regression was used to derive odds ratios for statistically significant associations. The level of significance was set at *p* < 0.05. Statistical analyses were performed using R—A language and environment for statistical computing (R Foundation for Statistical Computing, Vienna, Austria) [13].

## 3. Results

The study involved 4402 individuals, 2700 (61.3%) of them women and 1702 (38.7%) men, with a mean age of 53 and 54, respectively (SD 15.3, 15.8, respectively). Among the participants, 2878 (65.4%) were euthyroid, 773 (17.6%) were euthyroid with positive antibodies, 326 (7.4%) were subclinical hypothyroid, 131 (3%) were clinical hypothyroid, 47 (1.1%) were subclinical hyperthyroid and 7 (0.2%) were clinical hyperthyroid (Table 1). Table 1 includes descriptive statistics for all analysed parameters: gender, age, weight, thyroid hormones, Tg, positive TgAb and/or TPOAb antibodies, diagnosis, therapy and thyroid interfering drugs, both in the total sample and within each thyroid function group. Table 2 includes a detailed cross-tabulation overview of thyroid therapy intake and clinical thyroid diseases across thyroid function groups and previously established diagnoses. Women had a higher prevalence of euthyroidism with positive antibodies, subclinical hyperthyroidism, subclinical hypothyroidism and clinical hypothyroidism, while men had a higher prevalence of euthyroidism (Table 3). Out of the 2700 women in our study, 1049 were 50 years old or younger (with mean age 37.3 in the euthyroid, 36 and 34.4 in the clinically and subclinically hyperthyroid, 37 and 35.7 in the clinically and subclinically hypothyroid and 38 in the euthyroid with positive antibodies group).

Subclinical hypothyroid, clinical hypothyroid and subclinical hyperthyroid participants had a higher prevalence of positive antibodies, compared to the prevalence in the total sample (Table 4). Euthyroid with positive antibodies, clinical hyperthyroid, subclinical hyperthyroid and subclinical hypothyroid participants had a higher prevalence of previously established diagnosis of thyroid disorder, compared to the prevalence in the total sample (Table 4). Age followed an approximately normal distribution. Clinical hypothyroid participants were older than subclinical hypothyroid participants (Table 5). Weight was right-skewed due to the presence of outliers and extreme outliers which were true variable values and not errors in the data. There were no significant differences in weight distribution between thyroid function groups.

A logistic model with the male gender as a reference level revealed that women had 47% lower odds of euthyroidism than men. Additionally, women had 1.57 times higher odds of euthyroidism with positive antibodies, 2.1 times higher odds of subclinical hyperthyroidism, 2.37 times higher odds of clinical hypothyroidism and 1.58 times higher odds of subclinical hypothyroidism (Table 6).

Examining the presence of positive antibodies, we found that participants with positive antibodies had 2.01 times higher odds of subclinical hyperthyroidism, 4.3 times higher odds of clinical hypothyroidism, and 2.36 times higher odds of subclinical hypothyroidism than participants without positive antibodies (Table 6).

On the other hand, participants who were classified as euthyroid with positive antibodies had 2.26 times higher odds of being diagnosed, clinical hyperthyroid had 11.4 times higher odds of being diagnosed, subclinical hyperthyroid had 6.04 times higher odds of being diagnosed and subclinical hypothyroid had 2.47 times higher odds of being diagnosed than all other participants (Table 6).

## 4. Discussion

This is, to our knowledge, the first epidemiological study to analyse the prevalence of diagnosed and undiagnosed hypothyroidism, hyperthyroidism (subclinical and clinical) and positive thyroid antibodies in the iodine-sufficient Croatian population. The results of our study showed that the prevalence of clinical and subclinical hypothyroidism was 3% and 7.4%, respectively, while the prevalence of clinical and subclinical hyperthyroidism was 0.2% and 1.1%, respectively. Additionally, 17.6% of participants were euthyroid with positive antibodies. Most of these cases had not been previously diagnosed. The prevalence of undiagnosed subclinical and clinical hypothyroidism in our population was 6.9% and 2.8%, respectively, while the prevalence of undiagnosed subclinical and clinical hyperthyroidism was 0.9% and 0.1%, respectively. Thus, in our population, as much as 92.6% of subclinical hypothyroid, 93.9% of clinical hypothyroid, 83% of subclinical hyperthyroid, and 71.4% of clinical hyperthyroid participants were undiagnosed. Clinical hyperthyroid participants were most likely to be diagnosed (OR = 11.4). In addition to a higher prevalence of thyroid disorders, women were also more likely to develop antibody-positive euthyroidism, subclinical hyperthyroidism, clinical hypothyroidism and subclinical hypothyroidism. Men were more likely to be euthyroid. Participants with positive antibodies were more likely to develop subclinical hyperthyroidism, clinical hypothyroidism and subclinical hypothyroidism.

When we compared our results to the results of other studies, the prevalence of hypothyroidism was higher in our country than in most other countries, while the results for hyperthyroidism were quite similar. The first regulation on mandatory iodination of salt in Croatia was established in 1953 with the application of 10 mg of potassium iodide (KI) perkg of salt. Ten years later, a tenfold decrease in goitre was observed in the Croatian population. Research conducted between 1991 and 1993 showed a prevalence of goitre among school children between 8% and 35%. Consequently, 25 mg of KI per kg of salt was proposed and established in 1996 [14]. In 2009, a study conducted on school children showed iodine sufficiency in terms of urinary iodine concentration, thyroid volume and TSH levels, and therefore we can consider our population as iodine sufficient population [15]. When analysing the prevalence of hypothyroidism and hyperthyroidism in different countries, several aspects need to be considered. These include the influence of ethnicity, iodine intake, geographical location, the sensitivity of the assays used to detect TSH and thyroid hormone levels, and the lack of consensus on reference ranges [6,11]. Therefore, great variability in the prevalence of hypothyroidism and hyperthyroidism between countries has been observed. The largest thyroid function study conducted in the US, NHANES III, estimated that the prevalence of hypothyroidism was 4.6% (0.3% clinical and 4.3% subclinical), while the prevalence of hyperthyroidism was 1.3% (0.5% clinical and 0.7% subclinical) [5]. The US NHANES III study [5], and a study from Brazil [16], reported that individuals of African descent have a lower prevalence of hypothyroidism compared to Caucasians, confirming the strong influence of ethnicity on thyroid dysfunction. However, data on the prevalence of thyroid dysfunction in Africa are scarce due to a lack of population-based studies [3]. Compared to the NHANES III study, a study of the prevalence of thyroid disease in Colorado showed that the prevalence of hypothyroidism is higher in the US, with 8% of participants having subclinical hypothyroidism and 0.4% having overt hypothyroidism [17]. A recent US study found that the prevalence of undiagnosed subclinical and overt hypothyroidism was 6.06% and 0.82%, respectively, while the prevalence of undiagnosed subclinical and overt hyperthyroidism was 0.78% and 0.26%, respectively [11]. Although an increase in hypothyroidism has been reported in most countries implementing an iodination programme, hypothyroidism and hyperthyroidism could be caused by both excess iodine intake and iodine deficiency [3]. Studies have found both an increase [18,19], and a decrease [20,21] in the prevalence of hypothyroidism after implementing an iodination programme. To date, many studies have been conducted on the epidemiology of thyroid dysfunction in Europe. Two meta-analyses, one conducted in 2014 [6], and the other in 2019 [7], analysed the results of these studies. In a meta-analysis conducted by Garmendia Madariaga et al., it was observed that the prevalence of subclinical and overt hypothyroidism was 3.8% and 0.37%, respectively, while the prevalence of subclinical and overt hyperthyroidism was 2.91% and 0.68%, respectively [6]. This meta-analysis also showed that the prevalence of undiagnosed hypothyroidism was 4.94%, while the prevalence of undiagnosed hyperthyroidism was 1.72%. In a 2019 meta-analysis, Mendes et al. reported that the prevalence of hypothyroidism was lower among men than among women, with a higher prevalence of subclinical than clinical hypothyroidism in both genders [7]. In addition to the increased prevalence of hypothyroidism among women, Mendes et al. observed an increase in the prevalence of hypothyroidism with increasing age in Southern and Eastern Europe, compared to Northern and Western Europe. Most of the studies analysed in this meta-analysis (18 out of 21) had upper reference levels for TSH set at 4 mIU/L [7]. Only three studies had lower upper reference levels for TSH comparable to our study. Thus, two of these studies conducted in Germany and Norway found a lower prevalence of subclinical and clinical hypothyroidism than our study, although both used lower upper reference values for TSH (3.4 mIU/L and 3.5 mIU/L, respectively) [22,23]. In a third study, which included only 337 participants from Italy, the authors observed an extremely high prevalence of thyroid dysfunction: 12.5% of patients had subclinical hypothyroidism, 0.3% had overt hypothyroidism (upper reference level for TSH = 3.6 mIU/L), while 2.4% were affected by subclinical hyperthyroidism and 0.9% were affected by overt hyperthyroidism [24]. Such a high prevalence is probably the result of a very small sample size and low study power. Our study also had a very low upper reference level for TSH (3.6 mIU/L) compared to reference levels from other studies conducted in Europe [7]. We used the TSH reference range for our population (TSH: 0.3–3.6 mUI/L) [25]. Since the validation has not been performed in the Croatian population, the cut-off values for TSH used in Croatian clinics are those set by the assay manufacturer for the quantitative TSH test. The relatively higher prevalence of hypothyroidism detected in our population compared to other European countries could be partly due to the stricter upper reference level of TSH applied to our population. Another possible reason may lie in the fact that an increase in the prevalence of hypothyroidism has been reported in Southern Europe [7,24]. Although increased iodine intake is associated with an increased prevalence of hypothyroidism, iodine supplementation is encouraged because the benefits are considered to far outweigh the risks [26]. Potentially interfering drugs (amiodarone, oral steroids, corticosteroids, or estrogens) did not show an effect on thyroid function in our study because the largest number of participants taking such drugs (26/41) was observed in the euthyroid group.

Most studies, including our current study, have shown that the prevalence of hypothyroidism is higher in women [5,6,7,27]. Additionally, according to the results of our study, women were more likely to be antibody positive [5,11]. Many studies have shown that the prevalence of hypothyroidism increases with age [3,7,11]. Our current study showed that patients with clinical hypothyroidism were significantly older. However, subclinical hypothyroid patients were significantly younger. Although many studies have shown that body-mass index can affect TSH and thyroid hormone levels (reviewed in [28]), our current study did not observe an association between body weight and thyroid function groups.

The large sample size represents the strength of this study. A limitation of our study is its cross-sectional design, which made it impossible to take the complete status of the thyroid gland, i.e., at the time of participant recruitment, it was not possible to make a complete clinical examination and an ultrasound of the thyroid gland, so the recorded clinical diagnoses are mainly self-reported. Another limitation of the study is that it was performed in a middle-aged population (mean age 53, 53, 52, 56, 55 and 56 years in euthyroid, euthyroid with positive antibodies, subclinical, clinical hypothyroid, and subclinical and clinical hyperthyroid groups, respectively).

In conclusion, the correct diagnosis of thyroid dysfunction is extremely important due to the detrimental effects of thyroid dysfunction on human health (possible complications in patients with cardiovascular diseases and pregnant women). Our current study showed that a high proportion of thyroid dysfunction remained undiagnosed (as much as 92.6% subclinical hypothyroid, 93.9% clinical hypothyroid, 83% subclinical hyperthyroid, and 71.4% clinical hyperthyroid participants). Another important finding is the high prevalence of hypothyroidism among Croatians. Our study shows that we need to monitor all thyroid disorders because their impact on health depends on how early they are discovered. It is of most importance to invest in prevention programs because thyroid disorders are the leading endocrine disorders today.

## Figures and Tables

**Table 1:** Clinical characteristics of study participants.

	Total	Euthyroid	Euthyroid with Positive Antibodies	Clinical Hyperthyroid	Subclinical Hyperthyroid	Clinical Hypothyroid	Subclinical Hypothyroid
N (%)	4402	2878 (65.4%)	773 (17.6%)	7 (0.2%)	47 (1.1%)	131 (3%)	326 (7.4%)
Women (%)	2700 (61.3%)	1620 (56.3%)	539 (69.7%)	3 (42.9%)	36 (76.6%)	103 (78.6%)	231 (70.9%)
Age	53.4 (15.5)	53.2 (15.4)	53.6 (15.3)	56.71 (15.3)	55.7 (17.1)	56.72 (14.6)	52.02 (17.1)
Weight	77.3 (67.4, 88)	78 (67.4, 89.1)	76.1 (67.5, 86.1)	77.1 (67.8, 87.1)	76.3 (70.7, 87.3)	73.5 (65.3, 84.9)	75.1 (66.5, 86.5)
TSH	1.6 (1.1, 2.5)	1.47 (1.1, 2.1)	1.7 (1.2, 2.4)	0.04 (0.03, 0.22)	0.08 (0.03, 0.16)	5.78 (4.2, 8.5)	4.2 (3.9, 5.3)
fT3	4.4 (4.2, 4.8)	4.4 (4.3, 4.8)	4.4 (4.2, 4.8)	6.9 (6.1, 7.9)	5.3 (4.4, 6.2)	3.7 (3.3, 3.9)	4.3 (3.9, 4.7)
fT4	12.9 (11.9, 14.1)	13.1 (12.1, 14.1)	13 (12.1, 14.2)	25.2 (22.1, 27.1)	15.1 (12.7, 18.3)	9.9 (8.9, 10.1)	11.9 (10.9, 12.9)
Tg	9.7 (4.9, 15.9)	9.9 (5.4, 15.6)	8.9 (2.7, 16.2)	6.2 (0.8, 19.9)	8.1 (1.7, 21.5)	10.2 (4.9, 21.1)	10.5 (4.95, 16.8)
Positive TPOAb (%)	932 (21.1%)	0 (0%)	679 (87.8%)	4 (57.1%)	16 (34%)	67 (51.1%)	127 (39%)
Positive TgAb (%)	576 (13.1%)	0 (0%)	405 (52.4%)	2 (28.6%)	12 (25.5%)	46 (35.1%)	86 (26.4%)
Positive TPOAb and/or TgAb	1044 (23.7%)	0 (0%)	773 (100%)	4 (57.1%)	18 (38.3%)	73 (55.7%)	132 (40.5%)
Previously established diagnosis (%)	151 (3.4%)	0 (0%)	48 (6.2%)	2 (28.6%)	8 (17.02%)	8 (6.1%)	24 (7.4%)
Therapy (%)	99 (2.3%)	0 (0%)	35 (4.5%)	2 (28.6%)	7 (14.9%)	7 (5.3%)	17 (5.2%)
Thyroid interfering drugs	41 (0.9%)	26 (0.9%)	7 (0.9%)	0 (0%)	0 (0%)	1 (0.8%)	3 (0.9%)

Data are presented as mean (standard deviation), median (lower quartile, upper quartile) or as frequency (percentage). fT3, free triiodothyronine; fT4, free thyroxine; Tg, thyroglobulin; TgAb, thyroglobulin antibodies; TPOAb, thyroid peroxidase antibodies; TSH, thyroid-stimulating hormone.

**Table 2:** Distribution of thyroid therapy intake and clinical thyroid diseases across thyroid function groups and previously established diagnoses.

	Therapy	Euthyroid with Positive Antibodies	Clinical Hyperthyroid	Subclinical Hyperthyroid	Clinical Hypothyroid	Subclinical Hypothyroid
Diagnosis with subtotal/total thyroidectomy	Thyroid hormone replacement	3 (Graves’ disease/Hashimoto’s thyroiditis with nodular goitre)	0	1 (Graves’ disease)	0	1 (Nodular goitre)
	Antithyroid drugs	0	0	0	0	0
	No therapy	4 (Hashimoto’s thyroiditis with nodular goitre)	0	0	0	4 (Nodular goitre)
Diagnosis without thyroid surgery	Thyroid hormone replacement	32 (Hashimoto’s thyroiditis)	2 (after I-131 therapy/thyrotoxicosis factitia)	5 (after I-131 therapy)	7 (Hashimoto’s thyroiditis)	16 (Hashimoto’s thyroiditis)
	Antithyroid drugs	0	0	1 (Graves’ disease)	0	0
	No therapy	9 (Hashimoto’s thyroiditis)	0	1 (Graves’ disease)	1 (Hashimoto’s thyroiditis)	3 (Hashimoto’s thyroiditis)

Data are presented as frequency (diagnosis).

**Table 3:** Distribution of gender across thyroid function groups.

	Euthyroid	Euthyroid with Positive Antibodies	Clinical Hyperthyroid	Subclinical Hyperthyroid	Clinical Hypothyroid	Subclinical Hypothyroid
female	1620 (56.3%)	539 (69.7%)	3 (42.9%)	36 (76.6%)	103 (78.6%)	231 (70.9%)
male	1258 (43.7%)	234 (30.3%)	4 (57.1%)	11 (23.4%)	28 (21.4%)	95 (29.1%)
within female %	60%	20%	0.1%	1.3%	3.8%	8.6%
within male %	73.9%	13.7%	0.2%	0.6%	1.6%	5.6%
*p*-value	**<0.001 ^a^**	**<0.001 ^a^**	**0.441 ^b^**	**0.034 ^a^**	**<0.001 ^a^**	**<0.001 ^a^**

^a^ chi-square test, ^b^ Fisher’s exact test. Data are presented as frequency (percentage). Values in bold denote statistically significant *p*-values.

**Table 4:** Differences within thyroid function groups according to positive antibodies and previously established diagnosis, and prevalence of undiagnosed thyroid conditions.

Thyroid Function Group	Positive Antibodies			Previously Established Diagnosis			Prevalence of Undiagnosed Thyroid Conditions
	No	Yes	*p*-Value	No	Yes	*p*-Value	
Total	3358 (76.3%)	1044 (23.7%)		4251 (96.6%)	151 (3.4%)		
Euthyroid	2878 (100%)	0 (0%)		2878 (100%)	0 (0%)		
Euthyroid with positive antibodies	0 (0%)	773 (100%)		725 (93.8%)	48 (6.2%)	**<0.001 ^a^**	16.5%
Clinical hyperthyroid	3 (42.9%)	4 (57.1%)	0.058 ^b^	5 (71.4%)	2 (28.6%)	**0.022 ^b^**	0.1%
Subclinical hyperthyroid	29 (61.7%)	18 (38.3%)	**0.017 ^a^**	39 (83%)	8 (17%)	**<0.001 ^b^**	0.9%
Clinical hypothyroid	58 (44.3%)	73 (55.7%)	**<0.001 ^a^**	123 (93.9%)	8 (6.1%)	0.089 ^b^	2.8%
Subclinical hypothyroid	194 (59.5%)	132 (40.5%)	**<0.001 ^a^**	302 (92.6%)	24 (7.4%)	**<0.001 ^a^**	6.9%

^a^ chi-square test, ^b^ Fisher’s exact test. Data are presented as frequency (percentage). Values in bold denote statistically significant *p*-values.

**Table 5:** Age and weight characteristics across thyroid function groups.

Thyroid Function Group	Age				Weight	
	N	Mean (SD)	*p*-Value	N	Median (q1, q3)	*p*-Value
Total	4402	53.4 (15.5)	**0.041 ^a^**	4057	77.3 (67.4, 88)	0.134 ^b^
Euthyroid	2881	53.2 (15.4)		2797	78 (67.4, 89.1)	
Euthyroid with positive antibodies	770	53.6 (15.3)		758	76.1 (67.5, 86.1)	
Clinical hyperthyroid	7	56.7 (15.3)		6	77.1 (67.8, 87.05)	
Subclinical hyperthyroid	47	55.7 (17.1)		45	76.3 (70.7, 87.3)	
Clinical hypothyroid	131	56.7 (14.6) *		128	73.5 (65.3, 84.9)	
Subclinical hypothyroid	326	52.02 (17.1) *		323	75.1 (66.5, 86.5)	

^a^ ANOVA, ^b^ Kruskal–Wallis test, * indicate a statistically significant difference between the group means. Data are presented as mean (standard deviation) or median (lower quartile, upper quartile). Values in bold denote statistically significant *p*-values.

**Table 6:** Odds ratio (95% confidence interval) of variables significantly associated with thyroid function groups.

Thyroid Function Group	Gender	Positive Antibodies	Previously Established Diagnosis
	OR (95% CI)		
Euthyroid	0.53 (0.41, 0.68)		
Euthyroid with positive antibodies	1.57 (1.3, 1.85)		2.26 (1.59, 3.22)
Clinical hyperthyroid			11.4 (2.19, 59.23)
Subclinical hyperthyroid	2.1 (1.1, 4.1)	2.01 (1.1, 3.6)	6.04 (2.77, 13.2)
Clinical hypothyroid	2.37 (1.56, 3.62)	4.3 (3, 6.1)	
Subclinical hypothyroid	1.58 (1.24, 2.03)	2.36 (1.87, 2.98)	2.47 (1.57, 3.9)

Reference levels for logistic regression models: gender (male), positive antibodies (no), previously established diagnosis (group = no). Note that the dependent variable in the diagnosis model is the previously established diagnosis, and the independent variable is group belonging.

## Data Availability

Individual-level phenotypic data from CROATIA Split, Korcula and Vis cohorts are not available to outside researchers due to privacy restrictions.

## Abbreviations

fT3, free triiodothyronine; fT4, free thyroxine; KI, potassium iodide; NHANES III, The National Health and Nutrition Examination Survey; OR, odds ratio; SD, standard deviation; T4, thyroxine; Tg, thyroglobulin; TgAb, thyroglobulin antibodies; TPOAb, thyroid peroxidase antibodies; TSH, thyroid-stimulating hormone.
